# Effects of Astragalus injection and Salvia Miltiorrhiza injection on serum inflammatory markers in patients with stable coronary heart disease: a randomized controlled trial protocol

**DOI:** 10.1186/s13063-020-4109-6

**Published:** 2020-03-17

**Authors:** Zhihao Luo, Yuntao Liu, Zhen Zhao, Xia Yan, Dawei Wang, Qing Liu

**Affiliations:** 1 Guangdong Provincial Hospital of Chinese Medicine, The Second Clinical School of Medicine, Guangzhou University of Chinese Medicine, Guangzhou and Zhuhai, China; 2 Guangdong Provincial Key Laboratory of Research on Emergency in Traditional Chinese Medicine, Emergency Department, Guangdong Provincial Hospital of Chinese Medicine, Guangzhou, China; 3 Shunde Hospital of Guangzhou University of Chinese Medicine, Foshan, China; 4 Department of Immunology, School of Basic Medical Sciences, Fudan University, Shanghai, China

**Keywords:** Stable coronary heart disease, Astragalus injection, *Salvia miltiorrhiza* injection, Randomized controlled trial

## Abstract

**Background:**

Coronary heart disease (CHD) is a clinical syndrome caused by coronary atherosclerosis (AS) or functional changes in coronary arteries. We have previously reported that astragaloside IV (in astragalus) and tanshinone IIA (in *Salvia miltiorrhiza*) improve myocardial ischemic injury.

**Methods:**

This study will employ the randomized, blinded, prospective, single-center experiments approach. Briefly, a total of 160 eligible patients will be equally randomized into three treatment groups and placebo control groups. Patients will receive appropriate treatments every 24 h for seven days after enrollment and followed up for 28 days. The main evaluation indicators will be cell count, serum high-sensitivity C-reactive protein (hs-CRP) level, monocyte chemoattractant protein 1 (MCP-1), interleukin-6 (IL-6), interleukin-1β (IL-1β), interleukin-8 (IL-8), interleukin-18 (IL-18), interleukin-10 (IL-10), tumor necrosis factor (TNF)-α, oxidized low density lipoprotein (OX-LDL), angina grade, and Traditional Chinese Medicine (TCM) symptom changes scale. Secondary indicators to be evaluated will include B-type natriuretic peptide (BNP) levels, troponin (cTn), muscle enzyme isoenzyme (CK-MB), heart-type fatty acid binding protein (H-FABP), liver and renal functions, as well as blood coagulation. Close monitoring of adverse events during the trial will also be conducted.

**Discussion:**

This randomized controlled trial of Chinese herbal extracts for the treatment of coronary heart disease will generate results that are expected to provide valuable clinical evidence to inform future development of therapies towards management of CHD.

**Trial registration:**

China Clinical Trial Registration Center, ChiCTR1900021590. Registered on 28 February 2019.

## Background

Coronary heart disease (CHD) is a clinical syndrome caused by coronary atherosclerosis (AS) or functional changes in the coronary arteries. This condition results in the narrowing of the vascular lumen and insufficient blood supply to the coronary arteries, thereby increasing the load on the heart and leads to acute and temporary ischemia as well as hypoxia of the myocardium. Coronary AS is a serious disease that endangers human health. In recent years, studies have found that AS is not only a simple lipid deposit, but also an inflammatory disease. Local or systemic inflammatory response plays an important role in the development of AS and its related complications [1]. Therefore, anti-inflammatory therapies have been proposed as the ideal means of treating AS. In fact, many studies have shown that statins, angiotensin-converting enzyme inhibitors (ACEIs), antiplatelet agents, calcium channel blockers, and other drugs have certain anti-inflammatory properties that can be exploited to manage the condition [2].

Inflammatory factors, endogenous peptides mainly produced by immune cells with strong biological effects, are known to be indicators of inflammatory responses. Specifically, they mediate a variety of immune responses, which are closely related to occurrence and development of coronary heart disease [3]. Studies have shown that serum high-sensitivity C-reactive protein (hs-CRP) is one of the markers of inflammation, helping to indicate an increased risk of vascular events, and aid prognosis and risk stratification. Consequently, this marker is a potential target for the treatment of AS and its complications [4]. Numerous reports have demonstrated a close relationship between level of hs-CRP, monocyte chemoattractant protein 1 (MCP-1), interleukin-6 (IL-6), interleukin-8 (IL-8), and tumor necrosis factor (TNF)-α with pathogenesis of coronary heart disease [5, 6]. Since inflammatory factors are involved in the occurrence and development of CHD, exploring the relationship between various inflammatory factors and coronary heart disease can provide a deeper understanding of the pathogenesis of this disease. In addition, quantification of various inflammatory factors can help to dynamically understand a patient’s condition, while assessing the effects of treatment can guide clinical practice [7].

Coronary heart disease is categorized as “chest pain” and “heartache” in Traditional Chinese Medicine (TCM), which plays an important role in its diagnosis and treatment. In particular, the “Yiqihuoxue” is the most widely used method for managing the condition [8]. Among the components therein, astragalus and Salvia are the most commonly used compatibility drugs. In our previous studies, we focused on investigating the roles of TCM in CHD [9–12]. In particular, it is evident that astragalus membranaceus, the main component of astragalus membranaceus, and tanshinone IIA, the main component of *Salvia miltiorrhiza*, can effectively improve myocardial ischemic injury [13]. Functionally, the mechanism of action of the Salvia miltiorrhiza is related to inhibition of cell inflammatory reaction and apop-tosis in myocardial ischemic injury [14].

Based on the above understanding, we hypothesize, from a clinical perspective, that individual astragalus and *Salvia miltiorrhiza* injections, or a combination of the two, have certain anti-inflammatory properties and could play a role in the treatment of CHD. We therefore aim to explore the effects of *Salvia miltiorrhiza* treatment on stable CHD and provide the basis for its clinical application.

## Methods/design

### Study design and settings

**Fig. 1:**
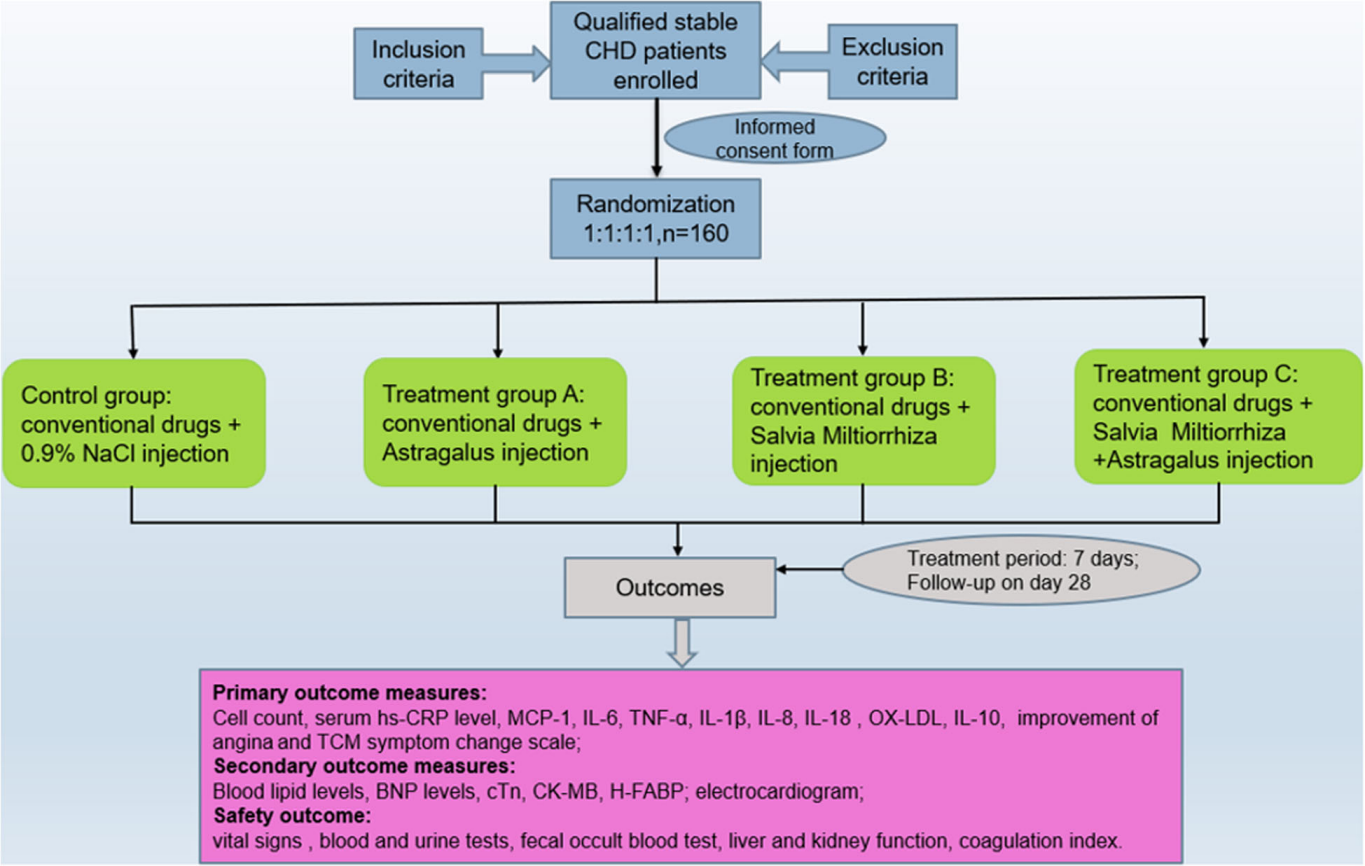
Trial flow chart

The present study will be set up using randomly blinded and single-center experiments. We will recruit 160 patients who meet the eligibility criteria at the Guangdong Provincial Hospital of Traditional Chinese Medicine. Subsequently, the participants will be divided into four equal groups: three groups with treatment (treatment group A will receive conventional drugs plus astragalus injection treatment, group B will receive conventional drugs plus *Salvia miltiorrhiza* injection, and group C will receive conventional drugs plus *Salvia miltiorrhiza* and astragalus injections) and the control group will receive conventional drugs plus 0.9% NaCl injection. The study design is as illustrated in Fig. 1.

### Objectives

This study intends to explore the effects of astragalus and *Salvia miltiorrhiza* injections as well as a synergistic treatment (of the two components) on stable CHD from the anti-inflammatory advantage and provide the basis for its clinical application.

### Research standard

All patients enrolled in the study should meet the following diagnostic and inclusion criteria. In addition, the patients will sign an informed consent form (ICF) before the study.

### Diagnostic criteria

The criteria for diagnosis of CHD will be as follows: (1) coronary angiography or coronary computed tomography (CT) examination confirmed at least one major branch lumen diameter stenosis of > 50%, clinical with or without angina, heart failure, arrhythmia, sudden or death recovery; (2) clear evidence of ST-segment elevation or non-ST-segment elevation myocardial infarction; and (3) a history of percutaneous coronary intervention (PCI) and/or coronary artery bypass grafting (CABG).

Clinical diagnostic criteria for chronic stable angina will be as described [15]: chronic stable angina is a condition in which a patient’s degree, frequency, nature, and predisposing factors of angina pectoris have not changed significantly within one month. The criteria will be as follows; (1) pain area: the typical part is the posterior sternum or left front chest, which can be radiated to the neck, pharynx, jaw, upper abdomen, shoulder and back, left arm, and left finger; (2) nature of pain: patients that often show symptoms of tightness, squeezing, pressure, burning, chest, chest tightness or suffocation, heavy feeling, subjective feelings vary widely, and some manifest as fatigue and shortness of breath; (3) duration: a paroxysmal episode lasting a few minutes, generally no more than 10 min; and (4) predisposing factors and mitigation methods: patients whose seizures are related to labor or emotional excitement can be relieved after stopping to rest and sublingual nitroglycerin can quickly relieve symptoms within 2–5 min.

### Inclusion criteria

The inclusion criteria are as follows: (1) patients meet the stable coronary atherosclerotic heart disease (SCAD) diagnostic criteria; (2) their medical history is > 3 months, frequency of angina pectoris is ≥ 3 times in the past one week, and the severity of angina pectoris is grade I–II/I–III; (3) patient’s age is 18–75 years; (4) patients with SCAD treated with any combination of cardiac-related drugs for > 3 months before enrollment, including atorvastatin, aspirin, angiotensin-II receptor blockers (ARBs), ACEIs, and beta-receptor blockers; and (5) patients signed the ICF.

### Exclusion criteria

The exclusion criteria are as follows: (1) patients with acute coronary syndrome (ACS) who are asymptomatic or have stable symptoms after stabilization; (2) patients requiring revascularization, including myocardial infarction, left ventricular dysfunction, multivessel disease, and/or large-scale myocardial ischemia (ischemic area > 10%), left main disease; (3) patients with chronic refractory angina pectoris; (4) patients with chronic kidney disease and severe heart, lung, liver, and other important organ dysfunction; serious primary diseases such as hematopoietic system and endocrine system, malignant tumor, gastrointestinal bleeding, gastric ulcer, and bleeding tendency are not expected complete the tester; (5) patients with severe and uncontrolled hypertension (systolic blood pressure ≥ 180 mmHg or diastolic blood pressure ≥ 110 mmHg); (6) those with severe arrhythmia (ventricular dichotomy, ventricular tachycardia, ventricular fibrillation, third degree atrioventricular block, arrest, severe sinus bradycardia, sick sinus syndrome, re-entrant supraventricular tachycardia, arrhythmia causing hemodynamic changes); (7) patients who participate in other clinical trials or are taking other proprietary Chinese medicines within one month; (8) pregnancy, or preparation for pregnancy, and lactating women; and (9) people with mental, psychological, and other problems that cannot be matched with the completion of the research.

### Loss to follow-up

Patients who are enrolling but have not completed clinical observations, including those withdrawing by themselves, losing their follow-up, poor compliance, or being withdrawn by a physician. If there are valid data in the case of shedding, the result of the last major efficacy index will be converted to the final result for statistical analysis.

### Suspension criteria

The trial will be suspended in case of the following: (1) serious adverse reactions occurred during the testing; and (2) the trial finds that the clinical trial program has major errors, or the program is good, but serious deviations occur during implementation, and it is difficult to evaluate the efficacy of the drug.

### Sample size

This study will use a parallel design of randomized controlled trials and hs-CRP as the main effect index. Conventional Western medicine treatment can reduce hs-CRP by 32%, according to clinical conditions, and it is estimated that Chinese medicine can reduce hs-CRP by 52%. The set α = 0.025, β = 0.2, superiority limit value δ = 20%, and the sample size of each group will be calculated as 30 cases [16, 17]. Considering the loss of follow-up, 40 patients with stable coronary heart disease and 160 with four combinations will be selected.

### Randomization

Random numbers will be generated using Statistics Analysis System (SAS) 9.2 software according to the principle of randomization. In order to implement random hiding, the random sequence will be kept by a trial manager. Using the remote random concealment method, it is determined that the basic information of qualified research individuals will be transferred by phone to the person who keeps the random numbers, from which the treatment allocation plan for each patient is obtained. Randomization of groups will be done according to a pre-made random scheme.

### Blinding

Due to the special color problem of the TCM injection, we anticipate that it will be difficult to achieve double-blinding during study. However, in order to avoid bias during the process, the following specific measures are proposed: (1) the researchers strictly adhere to the principle of randomization; (2) the doctors who participate in evaluation of the outcome are non-researchers, to ensure that evaluation of the efficacy of the test is blind; and (3) blood tests, data analysis, and statisticians to be covered up until the final analysis is completed. They only obtained the case report from (CRF) form and related data for analysis and did not understand the patient and drug use.

## Intervention

### Trial treatment methods

According to the principle of randomization, the group to be screened will be divided into four groups. The three groups of treatment are as follows: Group A, which receives conventional drug treatment plus astragalus injection treatment (size 20 mL/support, packaging 10/container), Group B, which will receive conventional medicine plus *Salvia miltiorrhiza* injection (specification 20 mL/support, packaging 10/container) and Group C, which will receive conventional medicine plus an injection of *Salvia miltiorrhiza* combined with astragalus. Both *Salvia Miltiorrhiza* and Astragalus injections will be prepared with 250 mL of 5% glucose. The control group receives an injection containing 250 mL conventional drug plus 5% glucose. For diabetic patients, an injection of 250 mL 5% glucose will be changed to 250 mL 0.9% NaCl. All patients will receive an intravenous drip once a day for 60 min for a treatment period of seven days.

The conventional drugs used in the stable coronary heart disease test will be as described in the ESC Guidelines for the diagnosis and treatment of SCAD [18]. The standard is given as aspirin, clopidogrel, low molecular weight heparin, statins, nitrates, beta-blockers, ACEIs, and other drugs.

### Pharmaceutical and combination methods

For allocation and combination of the drugs in this test, we will prepare *Salvia miltiorrhiza* injection and astragalus injection using 250 mL of 5% glucose. If the patient is diabetic, we will change 250 mL of 5% glucose injection into 250 mL of 0.9% NaCl. No other injections will be used simultaneously during intravenous infusion.

### Matters needing attention

During the study, patients will receive the test drug within 24 h of enrollment. Conventional treatment of stable coronary heart disease (such as anti-poly drug, lipid-lowering drug, antihypertensive drug) will be carried out at the same time. Injections of astragalus and *Salvia miltiorrhiza* should not be used as a substitute.

### Content and data capture points

The contents and key points of the data to be collected during the study include the screening period (one day), 24 h before recruitment, a treatment period of 1–7 days followed by daily recording of data, and follow-up on the 28th day after treatment.

## Outcome measures

### Primary outcome measures

Cell counts, serum detection by enzyme-linked immunosorbent assay, including serum hs-CRP level, MCP-1, IL-6, TNF-α, IL-1β, IL-8, IL-18, OX-LDL, IL-10, improvement of angina [19], and TCM symptom change scale [20] are outlined in Tables 1 and 2.

**Table 1:**
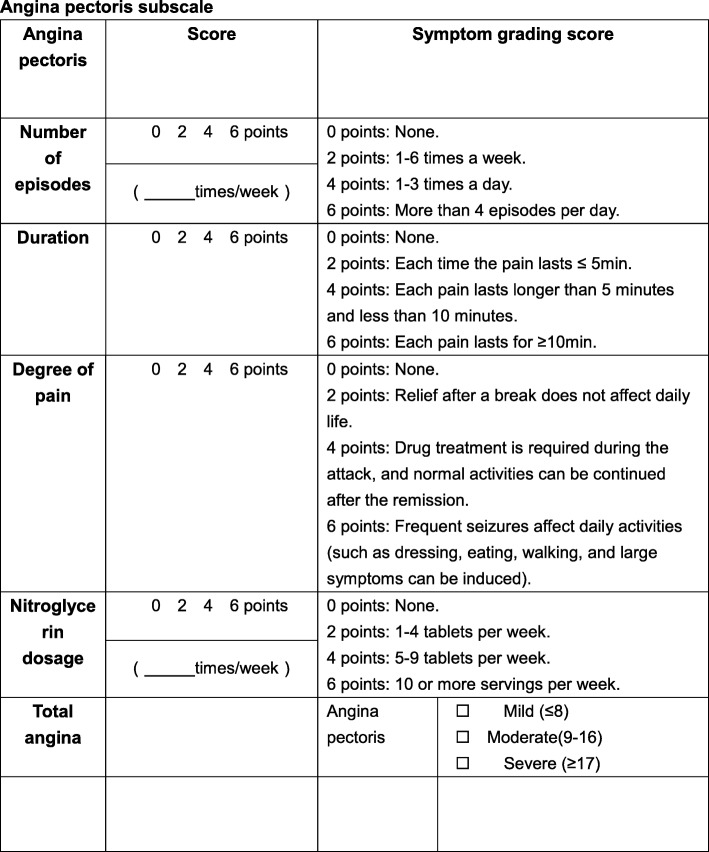
Angina pectoris subscale

**Table 2:** Coronary heart disease Chinese medicine main symptom scale

Symptom	Symptom grading score
**Chest pain**	☐ 0 points: None.
	☐ 3 points: Remission after rest will not affect daily life.
	☐ 6 points: medication is required at the time of onset, and normal life can be continued after remission.
	☐ 9 points: Frequent seizures affect daily activities (such as dressing, eating, walking, and large symptoms can be induced).
**Chest tightness**	☐ 0 points: None.
	☐ 3 points: I feel a chest tightness and can relieve myself.
	☐ 6 points: Chest tightness attacks are more frequent, but do not affect normal life and work.
	☐ 9 points: Chest tightness continues to be incomprehensible, affecting life and work.
**Shortness of breath**	☐ 0 points: None.
	☐ 2 points: short after the event.
	☐ 4 points: A little move is short.
	☐ 6 points: I usually feel short of breath.
**Weak**	☐ 0 points: None.
	☐ 2 points: Severe activity is weak.
	☐ 4 points: Moderate activity is weak.
	☐ 6 points: Mild activity is weak.
**Heart palpitations**	☐ 0 points: None.
	☐ 1 point: Occasionally, you can relieve yourself.
	☐ 2 points: Frequent attacks, but can persist at work.
	☐ 3 points: The heart is constantly puzzled, affecting life and work.

### Secondary outcome measures

The following measurements will also be taken; blood lipid levels; B-type natriuretic peptide (BNP); troponin (cTn); muscle enzyme isoenzyme (CK-MB); heart-type fatty acid binding protein (H-FABP); and electrocardiogram.

### Safety outcomes

These are vital signs including blood, urine, and fecal routines, liver and renal functions as well as coagulation. These indicators will be closely monitored during this process.

### Observation records of adverse events

Any adverse events that occur during the course of the trial, including laboratory abnormalities, must be carefully interrogated and tracked. We will therefore judge these events by their nature, severity, and drug relevance and strictly record their outcomes in the case report form.

### Research process record points

**Fig. 2:**
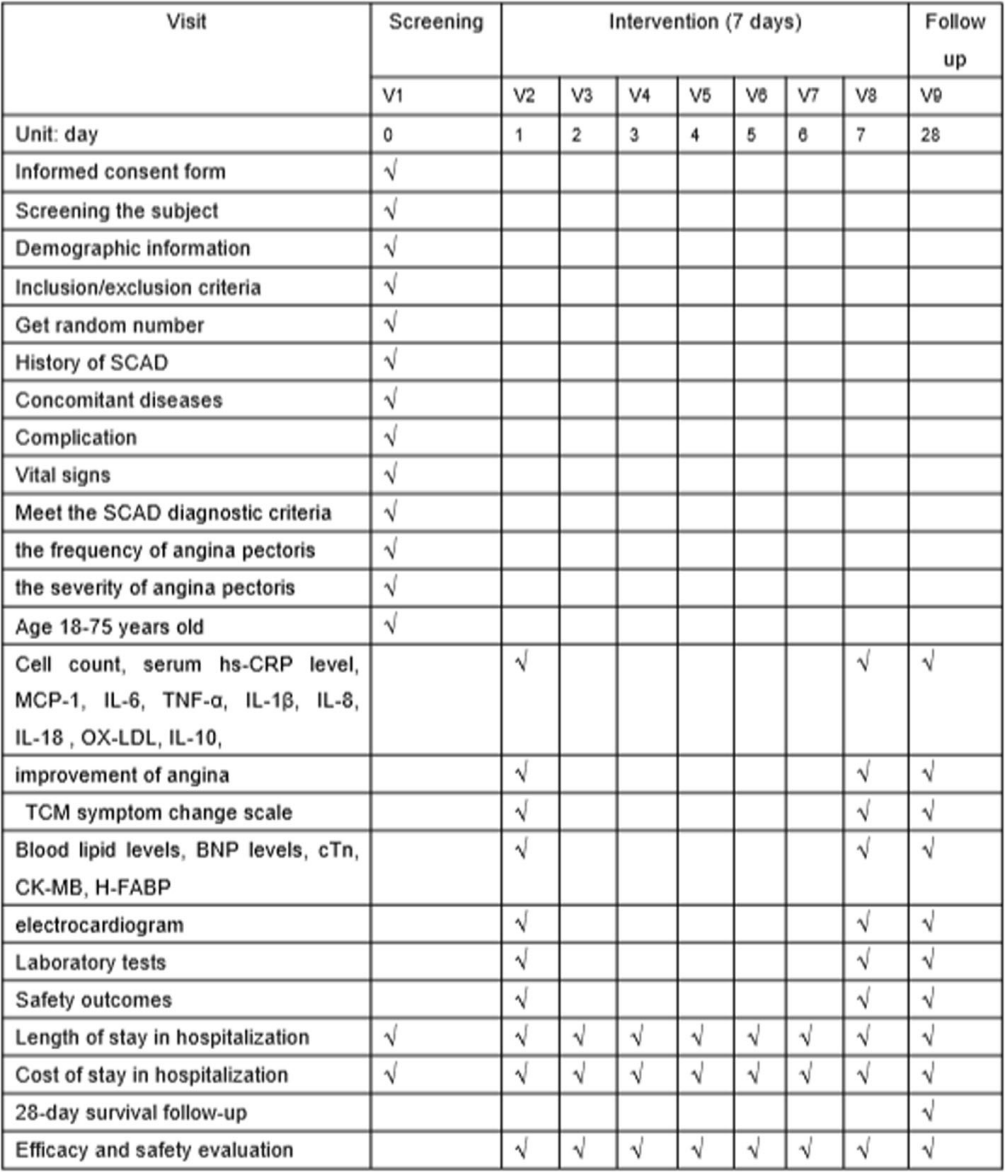
Content and points of data capture

The subject content and data at each time point, according to the patient’s hospitalization period, will be recorded as shown in Fig. 2. Specifically, the screening period (0 days) will be 24 h before recruitment followed by a daily data tracking and recording from day 1 to day 7, and a follow-up on day 28 after treatment is administered.

### Statistical analysis

Statistical analysis will be performed using SPSS 19.0 statistical software. Measured data will be described using means and standard deviation of the mean, while count data will be by the number of cases in percentages. First, a baseline analysis of the demographic characteristics in the four groups of selected cases will be conducted to investigate the balance and comparability across groups. Then, the effectiveness and safety indices will be compared across the four groups. The comparison of iso-quantitative data between the experimental and the control group will be performed using a student *t*-test of two-sample comparison for group design, while the comparison of the two classification indicators such as gender will be performed using the Cochran-Mantel-Haenszel χ^2^ test or Fisher’s exact probability method. The missing supplementary data will be adjusted using imputation methods. To validate ineffectiveness among the groups, we will perform a rank sum test of the two-sample comparison in the groups. All statistical tests will be two-sided with *p* ≤ 0.05 to be used for statistical significance.

### Data management

In the present study, the CRF will be filled out in time by the person in charge of the research. In addition, the patients will carefully fill in their relevant information and this will be regularly checked by clinical researchers to avoid missing data. The revisions provided by the clinical researcher will be implemented and corrected by the researcher in a timely manner. The researchers center will also confirm and sign the complete CRF form, in which no modifications are expected to be added after the trial. The entire research process will be conducted in a confidential manner, with none of the information disclosed to other parties. Only the lead researcher will have access to all data.

## Quality control

### Construction of research institutions and monitoring

During clinical research, multi-department construction and composition are required and each department should firmly cooperate to ensure the completion of quality research. In the current study, the main departments will include the following: (1) a competent department, composed of clinicians, pharmaceutical researchers, and quality supervision experts, which will be mainly responsible for the problems in clinical research, ensuring provision of solutions in a timely manner; (2) a Data Monitoring and Ethics Committee (DMEC): comprising experts in the field to monitor the overall conduct of the trial as well as relevant data and ensure that the rights of patients are adhered to; and (3) safety management: here, researchers from each center will be responsible for timely observations of the patient’s vital signs and laboratory indicators, and generate timely adjustments and feedback.

### Supervision and inspection

Effective supervision and inspection must be carried out throughout the clinical trial study. In this study, each researcher will conduct an examination once a week, including completion of a patient’s CRF, verifying a complete informed consent, accurate patient’s inclusion criteria, laboratory indicators and related scale collection, and preservation. During the test, patients with any adverse reactions and records of withdrawal will be identified. The researchers’ center will ensure authenticity and integrity of all data.

## Ethical issues

### Ethics statement

In clinical trials, researchers will comply with the Helsinki Declaration and ethical principles. The investigator will fully respect the individuals’ choices and will not force the individual to participate in the trial. The individual has the right to voluntarily participate, provide, and sign a written ICF, and may withdraw at any time. During recruitment, each participant will be assigned a random number to ensure blind implementation during the trial. In addition, researchers must protect the privacy and confidentiality of the individual.

### Ethical approval

This study has been approved by the Ethics Committee of the Guangdong Provincial Hospital of Traditional Chinese Medicine on 1 February 2019.A clinical research plan and the relevant documents of the ICF are approved by the Ethics Committee of the Guangdong Provincial Hospital of Traditional Chinese Medicine. The approval registration number is BF2018–182-01 (Table 3).

**Table 3:** Ethics committee names and approval registration number

Ethics committees’ name	Approval registration number
Ethics Committee of Guangdong Provincial Hospital of Traditional Chinese Medicine	BF2018–182-01

The hospital has approved qualification for drug clinical trials from the China Food and Drug Administration (CFDA) and the investigator had a Good Clinical Practice (GCP) certificate

### Informed consent form

The ICF will be fully explained to the individual or guardian by the researcher. Those agreeing to participate will sign the ICF, and both the researcher and subject will keep the signed ICF individually. Project managers will be allowed access to the ICF kept by researchers for monitoring and inspection.

## Discussion

CHD is a clinical syndrome caused by coronary AS or coronary functional changes. The condition results in the narrowing of the lumen in the blood vessels, insufficient blood supply to the coronary arteries, leading to cardiac load, and causing acute, transient ischemia as well as hypoxia of the myocardium [21]. AS is one of the main causes of coronary heart and cerebrovascular diseases, which contribute to a high global morbidity and mortality. Despite management options such as medical thrombolysis, and interventional or surgical bypass surgery, coronary artery recanalization directly results in myocardial ischemic injury. Myocardial ischemic injury has become a major clinical problem and has attracted wide research attention globally [22]. However, there is still no definitive and effective clinical method for prevention and treatment of this condition. It is, therefore, imperative to develop effective drugs for preventing and treating myocardial ischemic injury.

Recent studies have shown that chronic inflammation of the intima may play an important role in the pathogenesis of coronary atherosclerotic heart disease [16, 23]. There is a low degree of chronic inflammatory disease in AS. In fact, the vascular injury-response hypothesis suggests that endothelial dysfunction alters the anti-inflammatory properties of the endothelium. Inflammation, therefore, plays a major role in the development of atherothrombotic thrombosis and triggers cardiovascular events. For instance, since the 1990s, clinical research studies have linked cardiovascular events to chronic inflammation [24]. Furthermore, the role of inflammation in development of AS and sensitivity to predicting cardiovascular events has been identified. Current reports confirm that the inflammatory process is involved in the initiation of atherosclerotic progression and secondary lesions [25].

TCM has been vastly implicated in the prevention and treatment of CHD. In fact, since the development of integrated Chinese and Western medicine, coupled with the gradual deepening of research, TCM has acquired its own advantages in clinical practice enabling achievement of good efficacy. The basic pathogenesis of CHD is deficiency and blood stasis [22]. A treatment therapy for this condition has involved the use of *Salvia miltiorrhiza* injection, a TCM, whose main components are *Salvia miltiorrhiza* and antibalm. According to the relevant studies in modern medicine, *Salvia miltiorrhiza* can enhance cardiac function, improve the strength of myocardial contraction, increase blood supply to the myocardium, expand the blood vessels outside the heart, and generate very good anti-inflammatory effects [14]. In addition, *Salvia miltiorrhiza* injection has been found to promote blood circulation and remove blood stasis. Studies have further reported that static dripping of *Salvia miltiorrhiza* injection can significantly reduce inflammatory factors in patients with acute coronary syndrome [16], while in patients with acute myocardial infarction, its intravenous injection can reduce the level of hs-CRP and relieve chest tightness and pain [17]. Similarly, astragalus injection has been reported to increase myocardial contractility, with good protective effects on myocardial cells. Consequently, it has been found to effectively improve the cardiovascular function of patients. The drug can also strengthen the phagocytic function of macrophages, improve conversion rate of T cells, promote synthesis of interferon, and improve activity of interleukin. Furthermore, it affects immune regulation, antiviral replication, inhibits allergens, and bacteriostasis. Reports have shown that ulinastatin combined with astragalus injection generates good clinical effects on inflammatory reactions after PCI, which can reduce the expression levels of inflammatory factors in patients and reduce the myocardial injury caused by ischemia and reperfusion, which has important clinical application value [23].

In our previous studies, we found that the main components of astragalus, astragaloside IV, *Salvia viltiorrhiza*, and tanshinone IIA can effectively improve myocardial ischemic injury [13, 26]. Functionally, the mechanism of action of these drugs targeted cellular inflammation in myocardial ischemic injury and inhibited death. However, from a clinical point of view, it remains unclear whether individual astragalus and *Salvia miltiorrhiza* injections, or a combination of the two, result in significantly different anti-inflammatory properties, during the treatment of CHD. This study will explore the effects of the astragalus injection, the *Salvia miltiorrhiza* injection, and their synergistic treatment on stable CHD from the anti-inflammatory advantage, and provide the basis and basis for its clinical application.

### Trial status

Participant recruitment is currently underway. The version number / date is 2.0/20190601.

The start date of the recruitment of this project is 1 June 2019 and the approximate date of recruitment is 1 June 2020, for a period of one year.

## Data Availability

Not applicable.

## Abbreviations

ACEIs: Angiotensin-converting enzyme inhibitor
ACS: Acute coronary syndrome
ARBs: Angiotensin-II receptor blockers
AS: Atherosclerosis
ASA: Aspirin enteric capsules
BNP: B-type natriuretic peptide
CABG: Coronary artery bypass grafting
CHD: Coronary heart disease
CK-MB: Muscle enzyme isoenzyme
CRA: Clinical research associate
CRF: Case report form
cTn: Troponin
GCP: Good Clinical Practice
H-FABP: Heart-type fatty acid binding protein
hs-CRP: High-sensitivity C-reactive protein
ICF: Informed consent form
IL-10: Interleukin-10
IL-18: Interleukin-18
IL-1β: Interleukin-1β
IL-6: Interleukin-6
IL-8: Interleukin-8
MCP-1: Monocyte chemoattractant protein 1
OX-LDL: Oxidized low density lipoprotein
PCI: Percutaneous coronary intervention
PI: Principal investigator
SAS: Statistics Analysis System
SCAD: Stable coronary atherosclerotic heart disease
SFDA: State Food and Drug Administration
TCM: Traditional Chinese Medicine
TNF-α: Tumor necrosis factor
βRB: β- receptor blocker
